# Nek6 regulates autophagy through the mTOR signaling pathway to alleviate cerebral ischemia–reperfusion injury

**DOI:** 10.1186/s13041-024-01166-7

**Published:** 2024-12-19

**Authors:** Qingzhi Wang, Xinjing Liu, Jing Yuan, Ting Yang, Lan Ding, Bo Song, Yuming Xu

**Affiliations:** 1 Department of Neurology, The First Affiliated Hospital of Zhengzhou University, No. 1 Eastern Jian-She Road, Zhengzhou, 450052 Henan China; 2 Henan Key Laboratory of Cerebrovascular Diseases, The First Affiliated Hospital of Zhengzhou University, Zhengzhou, China; 3 NHC Key Laboratory of Prevention and Treatment of Cerebrovascular Disease, Zhengzhou, China

**Keywords:** Cerebral ischemia–reperfusion injury, Autophagy, Nek6, mTOR signaling pathway

## Abstract

**Objective:**

Cerebral ischemia–reperfusion injury (CIRI) is a major obstacle to neurological recovery after clinical treatment of ischemic stroke. The aim of this study was to investigate the molecular mechanism of Nek6 alleviating CIRI through autophagy after cerebral ischemia.

**Materials and methods:**

A mouse model of CIRI was constructed by middle cerebral artery occlusion (MCAO). TUNEL staining was used to observe the apoptosis of neuronal cells. The oxygen glucose deprivation/reoxygenation (OGD/R) model was established by hypoxia and reoxygenation. The cell apoptosis and activity was detected. Western blot was performed to detect the expression of autophagy-related proteins, protein kinase B (Akt)/mammalian target of rapamycin (mTOR) and adenosine 5’-monophosphate-activated protein kinase (AMPK)/mTOR signaling pathway-related proteins. Cellular autophagy flux was observed by fluorometric method. NIMA-related kinase 6 (Nek6) mRNA stability was detected by actinomycin D treatment. Methylation RNA immunoprecipitation technique was used to detect Nek6 methylation level.

**Results:**

Nek6 expression was increased in both MCAO and OGD/R models. Overexpression of Nek6 in OGD/R inhibited apoptosis, decreased LC3II and Beclin-1 expression, increased p62 expression, and occurred lysosome dysfunction. Interference with Nek6 has opposite results. Nek6 overexpression promoted p-Akt and p-mTOR protein expressions, inhibited p-AMPK and p-UNC-51-like kinase 1 protein expressions and cell apoptosis, while LY294002, Rapamycin or RSVA405 treatment reversed this effect. Abnormal methyltransferase·like protein 3 (METTL3) expression in CIRI enhanced m6A modification and promoted Nek6 expression level.

**Conclusion:**

This study confirmed that Nek6 regulates autophagy and alleviates CIRI through the mTOR signaling pathway, which provides a novel therapeutic strategy for patients with ischemic stroke in the future.

**Supplementary Information:**

The online version contains supplementary material available at 10.1186/s13041-024-01166-7.

## Introduction

Cerebral ischemia is a condition in which there is not enough blood in the brain to meet metabolic demands, which can lead to a lack of oxygen to the brain, resulting in an ischemic stroke [1, 2]. Ischemic stroke is one of the leading causes of death and disability in the population and have a complex pathogenesis. Cerebral ischemia–reperfusion injury (CIRI) is a phenomenon in which ischemic injury is further aggravated after occluded cerebral vessels are recanalized before brain tissue necrosis due to cerebral ischemia, which is an important pathophysiological basis for causing a variety of cerebrovascular diseases [3]. In the early stage of the disease, the electrical activity of local nerve cells stops, while the cell structure is basically intact, and the early restoration of blood supply can still restore neurological function. With the prolongation of ischemia, brain glial cells and local microvascular hyperplasia, which in turn leads to brain tissue edema, nerve cell degeneration and necrosis [4, 5]. Protecting the structure and function of nerve cells is an effective measure to promote the recovery of neurological function in stroke patients. Therefore, exploring the potential molecular mechanism of CIRI and developing new therapeutic targets to alleviate brain injury are the main objectives of current ischemic stroke research.

Autophagy is essential for neuronal homeostasis and function [6]. There is growing evidence that autophagy is impaired during cerebral ischemia, leading to neuronal dysfunction and neurodegeneration [7]. During cerebral ischemic injury, autophagy is detrimental to neurons, and inhibition of autophagy reduces infarct size and improves neurological scores [8]. Intracerebroventricular injection of Autophagy inhibitor (3-MA) pretreatment partially alleviated ischemia reperfusion-induced synaptic ultrastructural damage in middle cerebral artery occlusion (MCAO) mice [7]. Therefore, we need to investigate the mechanism of autophagy production in ischemic stroke in depth, which can provide potential targets for neuroprotection.

Through bioinformatics analysis of data set GSE93376 in Gene Expression Omnibus (GEO) database and comparison of gene differential expression in cerebral ischemia region of MCAO and control group, we found the candidate differential gene never in mitosis gene A (NIMA)-related kinase 6 (Nek6). NEK6 plays an important role in the progression of the mitotic cell cycle [9, 10]. It has been reported that Nek6 can accumulate abnormally under the condition of malregulated protein degradation mechanism, thus activating signal transducer and activator of transcription 3 to play an anti-apoptotic role, suggesting that Nek6 is closely related to cell growth and apoptosis [11]. Nek2, a member of the same family as Nek6, was reported to inhibit autophagy in gastric cancer via activating protein kinase B (Akt)/mammalian target of rapamycin (mTOR) signaling pathway [12]. Here, we speculated that Nek6 might affect autophagy through the mTOR pathway to alleviate MCAO-induced injury.

## Materials and methods

### Establishment of CIRI mice model by MCAO

All animal experiments are approved by the Ethics committee of The First Affiliated Hospital of Zhengzhou University. C57BL/6 J male mice (8–10 weeks, weight 23–25 g, Zhejiang Vital River Laboratory Animal Technology Co., Ltd.) were fasted for 12 h before surgery, and the MCAO model was established after anesthesia [13]. A 6-0 single-strand nylon wire was inserted from the bifurcation of the external carotid artery (ECA) or common carotid artery (CCA) into the internal carotid artery (ICA). The internal carotid artery was inserted to block the beginning of the middle cerebral artery (MCA) and all its blood supply, resulting in focal ischemia in the MCA supply area. After 1 h ischemia, the thread plug was withdrawn slowly. Only the ECA, CCA, ICA and MCA were separated, and the group without wire insertion was used as the sham group. Brain tissues from one mouse in the sham group (n = 7) and MCAO group (n = 7) was randomly selected for 2,3,5-triphenyltetrazolium chloride (TTC) staining, and single-cell suspension of peri-infarct brain tissues from the remaining 6 were taken for subsequent experiments.

### Intracerebroventricular injection

After MCAO establishment, the mice received one microinjection with AAV-Nek6 or AAV-NC (1 μL of 1 × 10^9^ viral genomes/μL) in the ipsilateral cortex ischemic lesions, and then grouped into MCAO + AAV-NC (n = 10), MCAO + AAV-Nek6 (n = 10). The coordinates of the microinjection were the following: 0.3 mm anterior to the bregma, 3 mm lateral, 2 mm deep and 1.9 mm posterior to the bregma, 3 mm lateral, 2 mm deep. The injection was completed with a 10 μL microsyringe at the rate of 0.1 μL/min, and the microinjector was placed for 10 min after injection. Brain tissues from one mouse in the MCAO + AAV-NC and MCAO + AAV-Nek6 group was randomly selected for TTC staining, and peri-infarct brain tissues from the remaining 4 were taken for subsequent experiments.

### Neurological deficit scores and TTC staining

Each group of mice was scored according to the modified neurological severity scores (mNSS). After scoring the neurological severity, the brains were removed by anesthesia and frozen at -20 °C for 20 min, then cut into 2 mm serial coronal sections in a brain tank and incubated with 0.2% TTC staining solution (Solarbio) for 30 min at 37 °C in a light-protected environment. The infarcted areas were pale white and the normal brain tissue was dark red after staining.

### TUNEL assay

Brain tissues from each group were dehydrated, embedded, sectioned and incubated in 0.9% NaCl injection for 5 min. Then the slices were incubated in TdT reaction mixture for 60 min at 37 °C. After washing with PBS, the tissues were blocked with 0.3% H_2_O_2_, 50 μL of TUNEL reaction mixture (Roche) was added, and incubated in the dark for 60 min at 37 °C in a humid environment. The sections were washed three times. The number of TUNEL-positive cells was observed under fluorescence microscope and photographed.

### Quantitative real-time polymerase chain reaction (QPCR)

Total RNA was extracted from single-cell suspension of peri-infarct brain tissues or SH-SY5Y cells using miR-Neasy Mini Kit (QIAGEN China (Shanghai) Co., Ltd.) and reverse transcribed into cDNA according to Taq Man MicroRNA Assays Reverse Transcription Primer instructions (QIAGEN China (Shanghai) Co., Ltd.). QPCR was performed according to SYBR Green PCR kit (Shanghai solarbio Bioscience Technology Co., LTD) instructions. β-actin was used as an internal reference and calculated using the 2^−△△Ct^ method.

### Western blot

Total protein from single-cell suspension of peri-infarct brain tissues or SH-SY5Y cells was extracted using RIPA lysis buffer (Beyotime), and protein concentrations were quantified by BCA Protein Assay Kit (Beyotime) according to the manufacturer's instructions. Proteins were separated by 8–10% sodium dodecyl sulfate–polyacrylamide gel electrophoresis (SDS-PAGE) and transferred to polyvinylidene difluoride (PVDF) membranes. After being closed in 5% skim milk powder for 1 h at room temperature, the membranes were incubated with anti-Nek6 (santa cruz, sc-374491), Beclin 1 (Abcam, ab207612), autophagy marker light chain 3B (LC3B, Abcam, ab221794), phospho-Akt (p-Akt, Ser473, cell signaling technology, #4060), Akt (cell signaling technology, #4685), phospho-mTOR (p-mTOR, Ser2448, cell signaling technology, #5536), mTOR (cell signaling technology, #2983), adenosine 5’-monophosphate-activated protein kinase α (AMPKα, cell signaling technology, #5831), p-AMPKα (Thr172, cell signaling technology, #50081), UNC-51-like kinase 1 (ULK1, Thermo Fisher Scientific, PA5-34542), p-ULK1 (Ser317, Thermo Fisher Scientific, PA5-104556), protein phosphatase 1α (PP1α, Thermo Fisher Scientific, 43-8100), p-PP1α (Thr320, Thermo Fisher Scientific, PA5-17819) at 4 °C overnight. The membranes were then incubated with secondary antibodies conjugated to HRP for 1 h at room temperature. The membranes were incubated for 1 h. The membranes were chromogenized using the EasyBlot ECL kit and the protein blot intensity was quantified by an Amersham Imager 600 system [14].

### Cell culture and establishment of oxygen glucose deprivation/reoxygenation (OGD/R) model

The neuronal cell line SH-SY5Y was provided by the Shanghai Cell Bank of the Chinese Academy of Sciences and cultured in DMEM medium containing 10% fetal bovine serum in a 5% CO_2_ incubator at 37 °C. SH-SY5Y cells were co-deprived of O_2_ and glucose for 1 h to simulate the in vitro injury model, after removing the original medium and adding sugar-free medium DMEM, and placed in a hypoxic chamber in saturated gas with 95% N_2_ and 5% CO_2_, and the oxygen concentration was maintained within 1%, and the temperature was controlled at 37 °C.

### Cell transfection and treatment

SH-SY5Y cells were inoculated in 6-well plates (1 × 10^5^/well) and transfected with pcDNA and pcDNA-Nek6, si-NC and si-Nek6, si-NC and si-methyltransferase·like protein 3 (si-METTL3) according to Lipofectamine 2000 reagent when the cell fusion level reached 70%. 12 h after transfection, fresh DEME medium containing fetal bovine serum was replaced and the cells were continued to be cultured for 48 h. The cells were collected for subsequent experiments. In the OGD/R + pcDNA-Nek6 + LY or OGD/R + pcDNA-Nek6 + Rapa group, cells were treated with 50 mmol/L LY294002 (LY, AKT inhibitor) or Rapamycin (Rapa, mTOR inhibitor) for 1 h before transfection. In the OGD/R + pcDNA-Nek6 + RSVA405 group, cells were treated with 3 μM RSVA405 (AMPK agonist) for 24 h. In the si-Nek6 + tautomycetin group, cells were treated with 20 nM PP1 inhibitor tautomycetin for 1 h.

### Cell apoptosis

Flow cytometry assay was performed to detect cell apoptosis. SH-SY5Y cells at logarithmic growth stage were inoculated with 1 × 10^4^/well in a 6-well plate and resuspended with complete culture medium. 5 μL Annexin V FITC and 5 μL PI was added for 15 min according to the instructions of the Annexin V-FITC/PI double-staining kit (Beyotime). Analysis was performed by flow cytometry within 30 mim.

### Cell activity

SH-SY5Y cells inoculated in 96-well plates were treated with OGD/R, 10 μL of MTT (5 g/L) solution was added and incubated for 4 h at 37 °C in a 5% CO_2_ incubator, and then 150 μL of DMSO was added and shaken for 10 min at room temperature. The absorbance value of each well at 490 nm was measured by using SynergyHT multifunctional microplate analyzer (Thermo Fisher Scientific).

### Tandem fluorescently labeled LC3 (mRFP-GFP-LC3) detects autophagy flux

SH-SY5Y cells were cotransfected with a tandem expression vector mCherry-GFP-LC3 (Shanghai Genechem Co., Ltd.) and pcDNA-Nek6 vector. OGD/R treatment was performed 24 h after transfection and observed by fluorescence microscope. EGFP (enhanced green fluorescent protein) is acid-sensitive and will lose its fluorescence when autophagosomes fuse with lysosomes, which reduces the pH value; whereas, mCherry is acid-insensitive and positively sustained in both autophagosomes and autolysosomes.

### Nek6 mRNA stability

Actinomycin D (ActD, Sigma) treatment was performed to detect the Nek6 mRNA stability. SH-SY5Y cells at logarithmic growth stage were treated with 5 μg/mL ActD, and total RNA of each group was extracted at 0, 1, 2, 3, and 4 h, respectively. Nek6 level was detected by qPCR.

### Prediction of methylation sites in Nek6 sequences

Login SRAMP website (https://www.cuilab.cn/sramp), enter Nek6 transcript sequence (fasta format), obtain Nek6 sequence of N6-methyl adenosine (m6A) methylation modification site.

### Methylated RNA immunoprecipitation (MeRIP)

Nek6 methylation level was detected by MeRIP assay by using a Magna RIP RNA-binding Protein Immunoprecipitation Kit (Millipore) in accordance with the manufacturer’s protocol. SH-SY5Y cells or tissues were lysed by RIP lysis buffer and incubated with antibodies against m6A (Synaptic Systems) at 4 °C overnight. IgG was used as negative control. The immunoprecipitated RNAs were eluted and analyzed by qPCR.

### Co-immunoprecipitation (Co-IP)

After the cells were washed with PBS, 500 μL RIPA lysate was added for lysis on ice, and centrifuged at 12,000 r/min for 5 min. 50 μL supernatant of lysate was taken as input group, and the remaining 450 μL supernatant was added to Nek6 antibody as IP group, and incubated overnight at 4 °C. The next day, 20 μL protein A/G magnetic beads (Thermo Fisher Scientific) were added and incubated at 4 °C for 2 h. After washing five times with wash Buffer, 50 μL of 1 × loading buffer was added for denaturation at 95 °C for 5 min. Western blot was used to determine the protein interaction between PP1 and Nek6.

### Statistical analyses

The cell experiment was repeated three times. SPSS 19.0 software was used for statistical analysis, and the measurement data was expressed as mean ± standard deviation (SD). Multi-group comparison was one way anova with tukey’s post-hoc, and two group comparison was unpaired two tailed t test. P < 0.05 was considered as statistically significant difference.

## Results

### Differential expression analysis of Nek6 protein in CIRI model

**Fig. 1:**
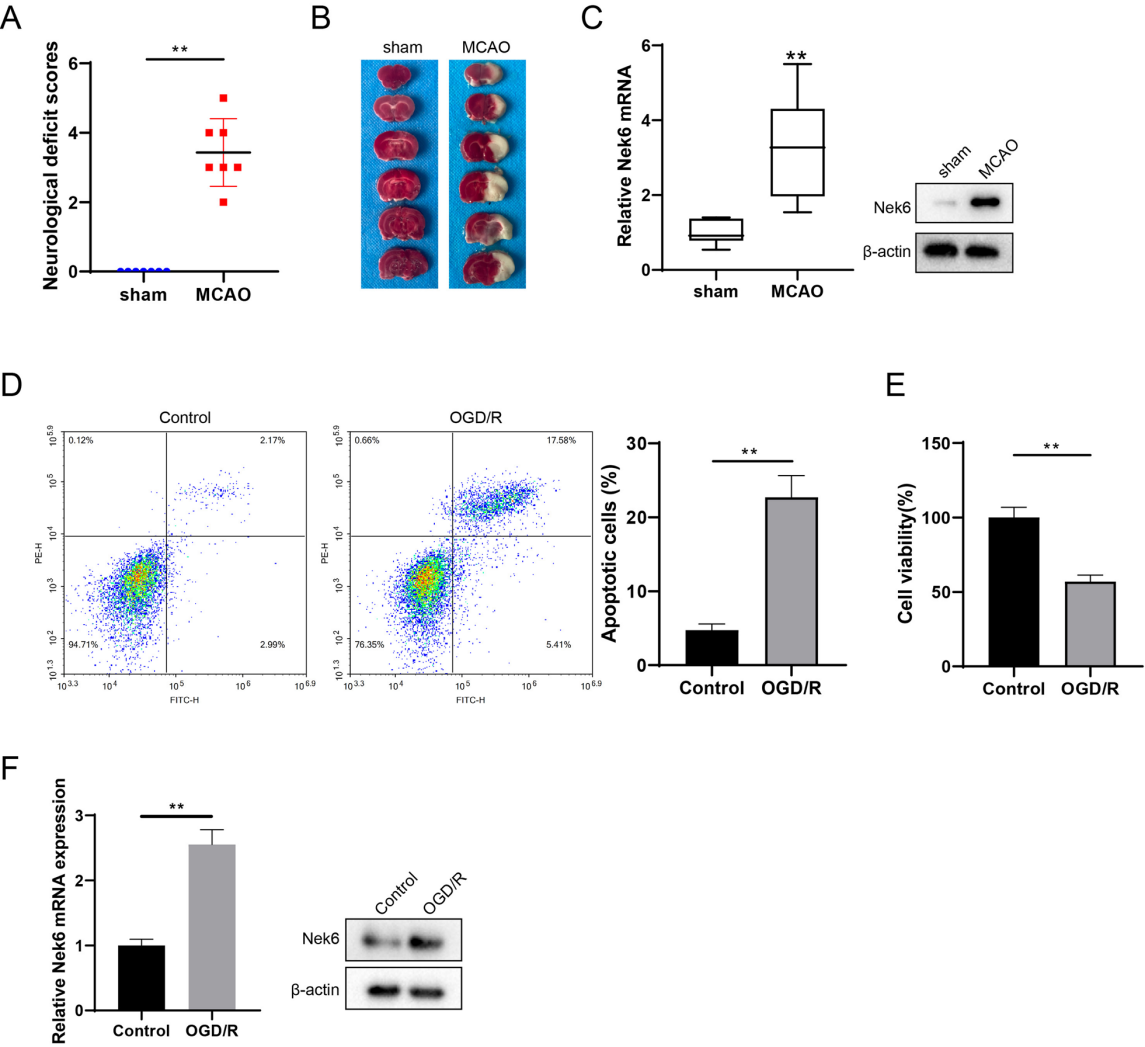
Differential expression analysis of Nek6 protein in CIRI model. The mice were divided into sham group and MCAO group. **A** Neurological deficit scores were evaluated in each group. N = 7. Difference was calculated using Mann Whitney test. **P < 0.01 vs*.* sham group. **B** TTC staining was used to detect the degree of cerebral infarct. N = 1. **C** The expressions of Nek6 in peri-infarct region were analyzed by qPCR and western blot. N = 6. Difference was calculated using unpaired two tailed T test. **P < 0.01 vs. sham group. **D–F** SH-SY5Y cells were grouped into control and OGD/R. **D** Cell apoptosis was detected by flow cytometry. **E** Cell activity was detected by MTT assay. **F** The levels of Nek6 mRNA and protein were detected by qPCR and western blot. Difference was calculated using unpaired two tailed T test. **P < 0.01 vs. control group

Firstly, a mouse model of CIRI was constructed by the MCAO method, and the neurological deficit scores were scored 24 h after successful modeling. Subsequently, TTC staining of brain tissues showed that the infarct volume in the MCAO group was increased compared with that in the sham group (Fig. 1A). To investigate the differential expression of genes in the cerebral ischemic region in the MCAO and sham groups of mice, we performed bioinformatics analysis on dataset GSE93376 in GEO database, and found that 62 genes were differentially expressed in the MCAO group (Figure S1A). These 62 genes were subjected to Gene Ontology functional annotation and six genes, Dab2, Lgals1, Nek6, Bcl2a1d, Pycard, and Lcn2, were found to be enriched to GO:0006915 ~ apoptotic process (Figure S1A). Notably, Lgals1, Bcl2a1d, and Lcn2 have been studied in ischemic stroke disease [15–17], so we focused on Dab2, Pycard, and Nek6 for validation. QPCR results showed that of the three differentially expressed genes, Nek6 showed the most significant mRNA changes in MCAO group (Figure S1B and 1C), and its protein expression was also increased (Fig. 1C). Combined with the analysis for Nek6, we next investigated the role of Nek6 in CIRI. Next, the neuronal cell line SH-SY5Y was grouped into control and OGD/R. The results in Fig. 1D and E showed increased cell apoptosis and decreased cell activity in the OGD/R group. QPCR and western blot assays revealed that Nek6 mRNA level and protein expression were up-regulated in the OGD/R group (Fig. 1F).

### Nek6 overexpression alleviated nerve injury and decreased autophagy and lysosome dysfunction in vitro

**Fig. 2:**
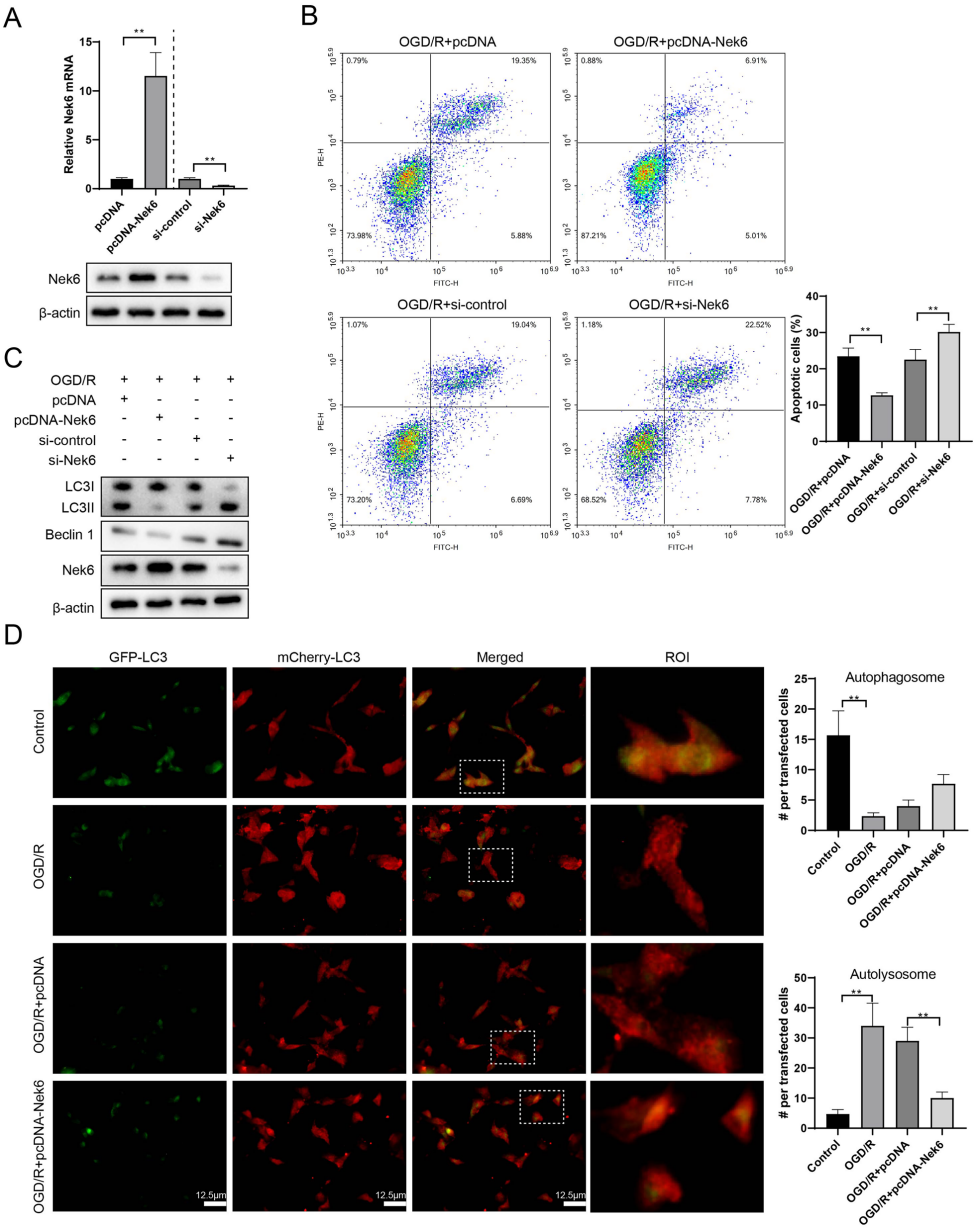
Nek6 overexpression alleviated nerve injury and decreased autophagy and lysosome dysfunction in vitro. **A**–**C** SH-SY5Y cells were grouped into OGD/R + pcDNA, OGD/R + pcDNA-Nek6, OGD/R + si-control, OGD/R + si-Nek6. **A** QPCR and Western blot were used to detect the expressions of Nek6. Difference was calculated using unpaired two tailed T test. **B** Cell apoptosis was detected by flow cytometry. Difference was calculated using one way anova with tukey’s post-hoc. **C** Western blot was used to detect the expressions of LC3 I/II, Beclin 1 and Nek6. **P < 0.01 vs. OGD/R + pcDNA or OGD/R + si-control. **D** SH-SY5Y cells were grouped into control, OGD/R, OGD/R + pcDNA, OGD/R + pcDNA-Nek6. Autophagy flux was detected by IF staining. Difference was calculated using one way anova with tukey’s post-hoc. **P < 0.01 vs. control or OGD/R + pcDNA

To investigate the effect of Nek6 on neuronal cells, SH-SY5Y cells was overexpressed or silenced with Nek6, followed by OGD/R treatment. Nek6 expression was increased after transfection with pcDNA-Nek6 and decreased after transfection with si-Nek6, indicating successful construction of the plasmids (Fig. 2A). Flow cytometry results revealed that overexpression of Nek6 significantly alleviated apoptosis induced by OGD/R treatment, while low expression of Nek6 was able to further promote apoptosis induced by OGD/R treatment (Fig. 2B). Since autophagy plays an important role in neuronal cell injury, we found elevated expression of LC3 II and Beclin 1 and decreased expression of sequestosome-1 (p62) protein in both in vivo and in vitro models, suggesting that CIRI can induce autophagy (Figure S2). In addition, pcDNA-Nek6 or si-Nek6 treatment had no effect on the expression of autophagy-related proteins under basal conditions (Figure S3). To further confirm the regulatory role of Nek6 on autophagy in the OGD/R in vitro model, we performed western blot assay on autophagy-related genes, and the results showed that overexpression of Nek6 decreased LC3 II and Beclin 1 expression in the OGD/R + pcDNA-Nek6 group, and interfering with Nek6 had the opposite effect in the OGD/R + si-Nek6 group (Fig. 2C). Red puncta in merged photos can characterize autolysomes, and double staining puncta can characterize autophagosomes. Autophagy flux identification experiment results showed that OGD/R treatment showed more autolysosomes, while Nek6 overexpression significantly reduced autolysosomes (Fig. 2D). Combined with western blot detection, we conclude that Nek6 inhibits the formation of autolysosomes.

### Nek6 inhibits autophagy through the Akt/mTOR and AMPK/mTOR pathway

**Fig. 3:**
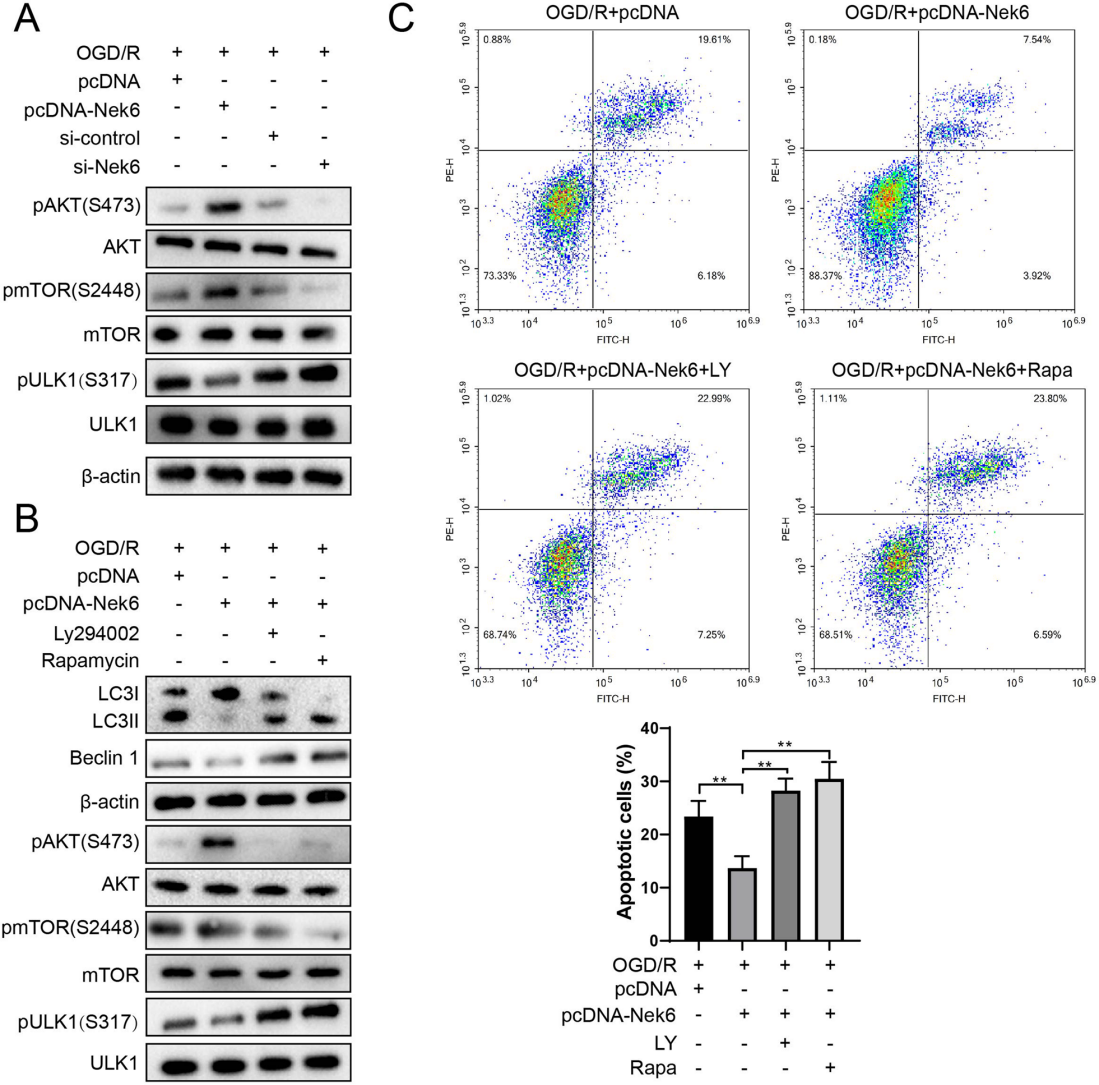
Nek6 inhibits autophagy through the Akt/mTOR pathway. **A** SH-SY5Y cells were grouped into OGD/R + pcDNA, OGD/R + pcDNA-Nek6, OGD/R + si-control, OGD/R + si-Nek6. Western blot was used to detect the expressions of p-Akt, Akt, p-mTOR, mTOR, ULK1 and p-ULK1. **B**, **C** SH-SY5Y cells were grouped into OGD/R + pcDNA, OGD/R + pcDNA-Nek6, OGD/R + pcDNA-Nek6 + LY and OGD/R + pcDNA-Nek6 + Rapa. **B** Western blot was used to detect the expressions of LC3 I/II, Beclin 1, p-Akt, Akt, p-mTOR, mTOR, ULK1 and p-ULK1. **C** Cell apoptosis was detected by flow cytometry. Difference was calculated using one way anova with tukey’s post-hoc. **P < 0.01 vs. OGD/R + pcDNA or OGD/R + pcDNA-Nek6

Nek2, which belongs to the same family as Nek6, has been reported to inhibit autophagy in gastric cancer via activating AKT signaling pathway [12]. In addition, it is known that negative regulation pathway Akt/mTOR and positive regulation pathway AMPK/mTOR are important for autophagy regulation [18, 19]. Therefore, we next explored the effect of Nek6 regulation of Akt/mTOR signaling pathway on autophagy. In the Fig. 3A and Figure S4A, higher p-Akt and p-mTOR protein expressions, and lower p-AMPK and p-ULK1 protein expressions in the Nek6 overexpression group were observed, and the opposite result was observed in the si-Nek6 group, indicating that Nek6 can promote the activation of mTOR signaling pathway after OGD/R. When Nek6 overexpression cells were treated with LY294002 (an AKT inhibitor), Rapamycin (a mTOR inhibitor) or RSVA405 (AMPK agonist), it not only reversed the activation of AKT/mTOR signaling pathway and inhibition of AMPK/mTOR signaling pathway by Nek6, but also promoted the expression of p-ULK1, LC3 II and Beclin 1 (Fig. 3B and Figure S4B). Studies have confirmed that direct binding of NeK2-based PP1 phosphorylates the Thr320 site of PP1, which leads to up-regulation of p-AKT (S473) [20]. Therefore, it is speculated that Nek6 may also activate the AKT pathway by binding PP1, and Co-IP assay found that Nek6 could bind to PP1 (Figure S5A), and Western blot detection found that overexpression of Nek6 promoted PP1 phosphorylation (T320), while knockdown of Nek6 inhibited p-PP1 (Figure S5B). Furthermore, treatment with PP1 specific inhibitor tautomycetin abolished the inhibition of AKT and mTOR phosphorylation by interfering with Nek6, that is, Nek6 activated the downstream AKT/mTOR pathway by binding and inactivating PP1. Furthermore, treatment with PP1 specific inhibitor tautomycetin abolished the inhibition of AKT and mTOR phosphorylation by interfering with Nek6, that is, Nek6 activated the downstream AKT/mTOR pathway by inactivating PP1 (Figure S5C). In addition, compared with the OGD/R + pcDNA group, apoptotic cells in the Nek6 overexpression group were significantly decreased, and LY294002, Rapamycin or RSVA405 treatments could reverse the apoptotic effect of Nek6 (Fig. 3C and Figure S3C). These results suggest that Nek6 inhibits autophagy and alleviates nerve injury through Akt/mTOR and AMPK/mTOR pathway.

### Nek6 overexpression alleviated nerve injury after MCAO in vivo

**Fig. 4:**
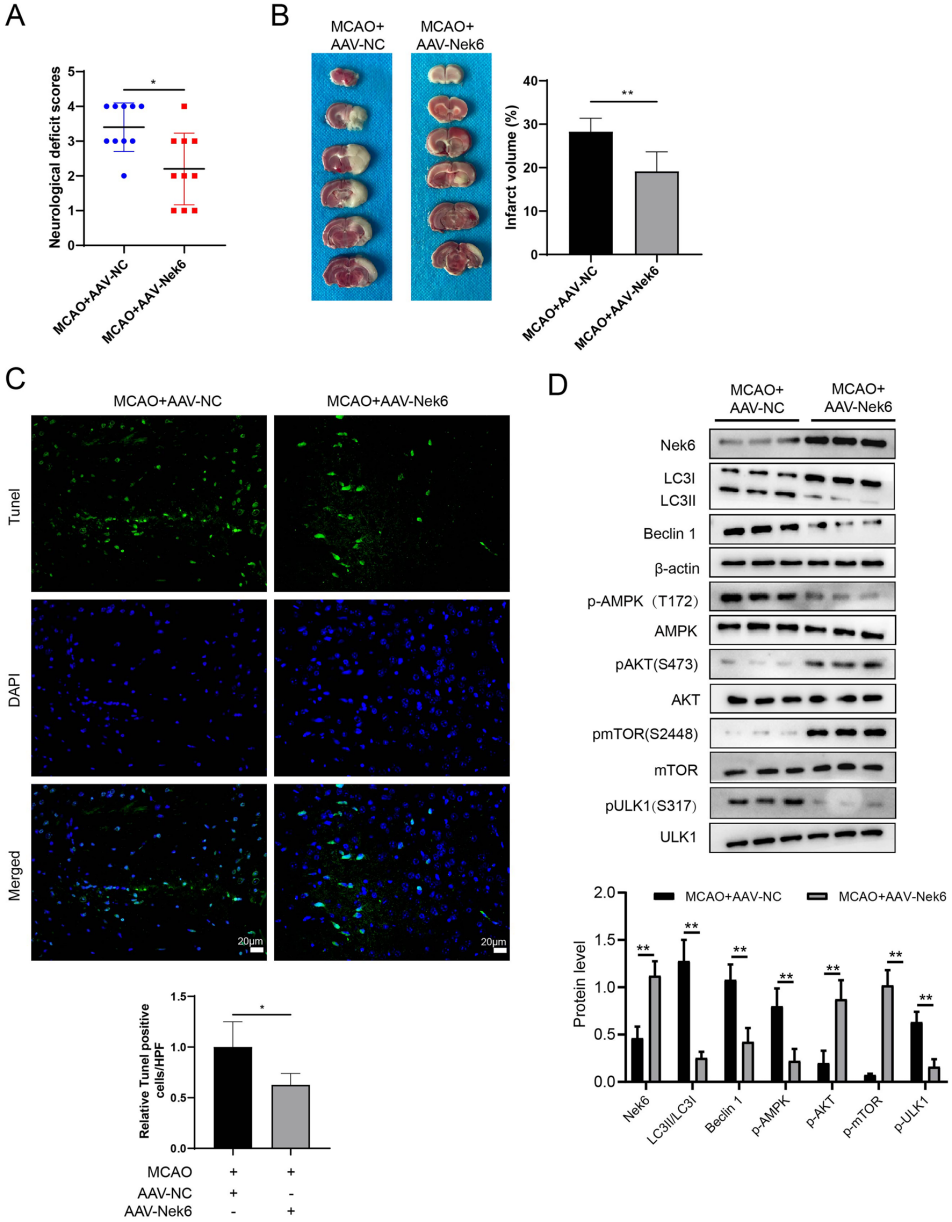
Nek6 overexpression alleviated nerve injury after MCAO in vivo. The mice were divided into MCAO + AAV-NC and MCAO + AAV-Nek6. **A** Neurological deficit scores were evaluated in each group. N = 10. Difference was calculated using mann whitney test. **B** TTC staining was used to detect the degree of cerebral infarct. N = 6. **C** Cell apoptosis was detected by TUNEL assay. N = 4. **D** The expressions of LC3 I/II, Beclin 1, p-Akt, Akt, p-mTOR, mTOR, p-AMPK, AMPK, ULK1 and p-ULK1 in peri-infarct region were analyzed by western blot. N = 4. **B**–**D** Difference was calculated using unpaired two tailed T test. *P < 0.05, **P < 0.01 or P < 0.001 vs. MCAO + AAV-NC

In order to verify the effect of Nek6 on CIRI in vivo, we detceted neurological deficit scores in the MCAO + AAV-NC and MCAO + AAV-Nek6 groups of mice, and found that the neurological deficit scores were reduced after overexpression of Nek6 (Fig. 4A). The results of TTC staining showed a reduction in the area of brain infarction (Fig. 4B). Next, TUNEL and western blot were performed on the peri-infarct region. The results in Fig. 4C showed that AAV-Nek6 injection could significantly reduce the apoptosis of nerve cells and alleviate MCAO injury. Furthermore, Nek6 overexpression in vivo decreased the expressions of LC3 II and Beclin 1, and increased the protein expressions of p-Akt, p-mTOR, p-AMPK and p-ULK1, indicating that Akt/mTOR signaling pathway was enhanced and AMPK/mTOR signaling pathway was weakened, which leading to the inhibition of autophagy (Fig. 4D).

### Nek6 mRNA was post-transcriptional regulated through m6A modification

**Fig. 5:**
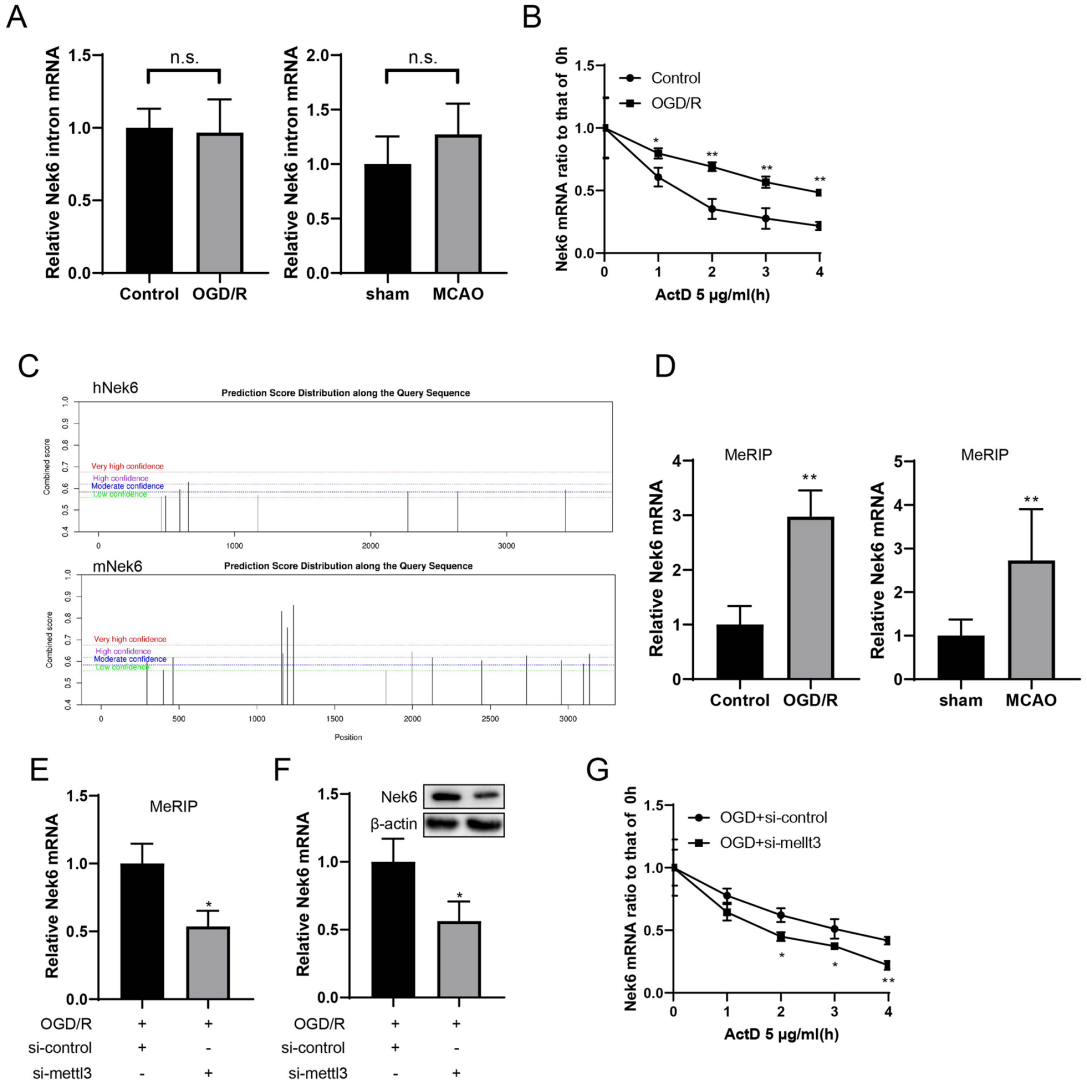
Nek6 mRNA was post-transcriptional regulated through m6A modification. **A** The expressions of Nek6 introns in peri-infarct region of mice (N = 6) or SH-SY5Y cells were analyzed by qPCR. **B** ActD treatment was used to evaluate Nek6 mRNA stability. **C** The m6A binding site of Nek6 mRNA was predicted by SRAMP. **D** MeRIP and qPCR were used to detect Nek6 methylation level in vivo (N = 6) and in vitro. *P < 0.05, **P < 0.01 vs. sham group or control group. n.s. means no significance. **E** SH-SY5Y cells were grouped into OGD/R + si-control, OGD/R + si-METTL3. MeRIP and qPCR were used to detect Nek6 methylation level. **F** QPCR and Western blot were used to detect the expressions of Nek6. **G** ActD treatment was used to evaluate Nek6 mRNA stability. **A**, **B**, **D–G** Difference was calculated using unpaired two tailed T test. *P < 0.05, **P < 0.01 vs. OGD/R

To analyze how OGD/R contribute to Nek6 mRNA upregulation, we examined the expression of Nek6 introns, which are involved in premature RNAs (pre-mRNA). Intron levels of Nek6 was not significantly altered by OGD/R or MCAO treatment (Fig. 5A), indicating that Nek6 mRNA might be up-regulated through post-transcriptional way. After OGD/R and control group cells were treated with ActD, it was found that Nek6 mRNA was degraded slowly in the OGD/R group (Fig. 5B). Based on this result, we confirmed that changes in Nek6 mRNA levels was post-transcriptional regulated. The SRAMP site predicted that Nek6 mRNA had more m6A binding sites (Fig. 5C). Next, MeRIP assay combined with qPCR test showed that the degree of Nek6 m6A modification in both in vivo and in vitro model group was significantly higher than that in control group (Fig. 5D). After METTL3 silencing, Nek6 methylation level was decreased (Fig. 5E), mRNA and protein expressions of Nek6 were also decreased (Fig. 5F). In addition, after ActD treatment, the degradation rate of Nek6 mRNA in OGD/R + si-METTL3 group was accelerated (Fig. 5G), which fully indicated that m6A modification of Nek6 could promote the stability of its mRNA.

## Discussion

Autophagy has dual effects on cerebral ischemia–reperfusion. Yuan et al*.* found that activating autophagy can protect the brain against ischemia–reperfusion injury [21], but Feng et al*.* found that inhibiting autophagy can alleviate acute neuronal injury after ischemic stroke [8]. Therefore, the present study aimed to explore the molecular mechanism by which Nek6 alleviates CIRI through autophagy. The occurrence and development of autophagy requires the involvement of many genes and proteins, and the expression levels of multiple autophagy-related proteins and the pathway activity of autophagy-related signals are increasingly being considered for the future treatment of ischemic stroke. Beclin1, LC3 and p62 are all signature proteins for the detection of autophagic activity [22]. Studies have confirmed that the expression of autophagy proteins Beclin1 and LC3 are up-regulated after CIRI, suggesting that CIRI can activate the body’s own autophagy level, which further aggravates neuronal damage in ischemic brain tissue [23]. The results of the present study also found that the levels of autophagy factors Beclin-1 and LC3-II protein were elevated in the model group, while the levels of p62 protein were decreased, which is consistent with the above study reports. ULK1, as an initiating kinase that regulates autophagy in mammals, can be phosphorylated by mTOR and AMPK upstream and transmit these signals to downstream mediators to balance autophagy regulation [24]. In addition, overexpression of Nek6 in vivo or in vitro CIRI model could reduce LC3 II, Beclin 1 and p-ULK1 expressions and exert an inhibitory effect on autophagy.

Autophagy is regulated by several signaling pathways, among which PI3K/Akt/mTOR signaling pathway is one of the most widely studied signaling pathways [25]. Akt is an important inhibitor of apoptosis and is phosphorylated to p-Akt upon signal activation, which is a signaling pathway positive activation marker. mTOR is an important transduction molecule downstream of Akt [26], and regulate cellular autophagy inhibitory genes. mTOR phosphorylation to p-mTOR mediates the downstream molecules of the Akt/mTOR pathway to reduce autophagic activity, inhibit apoptosis, and promote cell cycle progression [27–29]. In addition, mTOR is also a downstream protein of AMPK, and AMPK/mTOR signaling pathway plays an important role in cell growth, cell proliferation, energy metabolism and autophagy regulation [30]. Here, overexpression of Nek6 in vivo or in vitro CIRI model activates the mTOR signaling pathway and inhibits autophagy. In vitro experiments revealed that treatment with LY294002 (AKT inhibitor), Rapamycin (mTOR inhibitor) or RSVA405 (AMPK agonist) reversed the inhibitory effect of Nek6 overexpression on autophagy-related molecules and the mitigating effect of apoptosis, suggesting that Nek6 alleviates neural injury after CIRI through the AKT/mTOR pathway and AMPK/mTOR pathway.

mRNA degradation is a post-transcriptional mechanism that regulates mRNA abundance, of which m6A is one of the most frequently studied modalities of mRNA degradation and has important implications for mRNA stability [31–33]. m6A methylation modification is one of the most common epigenetic modifications of eukaryotic mRNA, accounting for more than 50% of all RNA methylation [34, 35]. Recent studies have shown that changes in m6A modification and related proteins can cause nervous system dysfunction and play a key role in the development of many neurological diseases [36]. Recently, Chang et al*.* showed that brain ischemia alters m6A methylation levels, and m6A methylated modified RNAs are involved in pathophysiological processes after ischemic stroke, including CIRI, inflammation, oxidative stress and apoptosis [37]. m6A dynamic chemical modification process is mainly regulated by related enzymes, among which METTL3 is the most important component protein of m6A methyltransferase complex. Few studies have focused on the potential mechanisms of METTL3 in CIRI occurrence, so this study investigated whether there is a regulatory role of METTL3 on Nek6 in CIRI. Here, both in vivo and in vitro experiments revealed a higher degree of Nek6 m6A modification in the model group. In vitro construction of a METTL3 low expression model revealed reduced m6A modification of Nek6, reduced mRNA and protein expression, and reduced stability of its mRNA.

In summary, our results suggest that m6A methylated Nek6 is involved in the pathophysiology of CIRI by affecting the mTOR signaling pathway and thereby regulating autophagy, which provides novel insights into the therapeutic mechanism of CIRI.

## Supplementary Information

Supplementary Material 1. Fig. 1 (A) Bioinformatics analysis was performed on dataset GSE93376 in GEO database. 62 differentially expressed genes were subjected to Gene Ontology functional annotation. B QPCR was used to detect the levels of Dab2 and Pycard. N = 6. Difference was calculated using unpaired two tailed T test. **P < 0.01 vs. sham group.

Supplementary Material 2. Fig. 2 Western blot was used to detect the expressions of LC3 I/II, Beclin 1 and p62 in vivo (A) and in vitro (B).

Supplementary Material 3. Fig. 3 (A) SH-SY5Y cells were grouped into pcDNA, pcDNA-Nek6, si-control, si-Nek6. Western blot was used to detect the expressions of LC3 I/II, Beclin 1 and p62. Difference was calculated using unpaired two tailed T test. ns: no significance.

Supplementary Material 4. Fig. 4 (A) SH-SY5Y cells were grouped into OGD/R + pcDNA, OGD/R + pcDNA-Nek6, OGD/R + si-control, OGD/R + si-Nek6. Western blot was used to detect the expressions of p-AMPK and AMPK. (B-C) SH-SY5Y cells were grouped into OGD/R + pcDNA, OGD/R + pcDNA-Nek6, and OGD/R + pcDNA-Nek6 + RSVA405. (B) Western blot was used to detect the expressions of LC3 I/II, Beclin 1, p-AMPK, AMPK, p-mTOR, mTOR, ULK1 and p-ULK1. (C) Cell apoptosis was detected by flow cytometry. Difference was calculated using one way anova with tukey’s post-hoc. **P < 0.01 vs. OGD/R + pcDNA or OGD/R + pcDNA-Nek6.

Supplementary Material 5. Fig. 5 (A) The interaction between Nek6 and PP1 was validated by Co-IP assay. (B) Western blot was used to detect the expressions of PP1α, p-PP1α (T320) in the pcDNA, pcDNA-Nek6, si-control, si-Nek6 groups. (B) Western blot was used to detect the expressions of p-AMPK, AMPK, p-mTOR, mTOR in the si-control, si-Nek6, si-Nek6 + tautomycetin groups.

## Data Availability

The datasets used and/or analyzed during the current study are available from the corresponding author on reasonable request.
