# Supplementary figures and images for: Nek6 regulates autophagy through the mTOR signaling pathway to alleviate cerebral ischemia–reperfusion injury
Source: Mol Brain. 2024 Dec 19;17:96. doi: 10.1186/s13041-024-01166-7 (PMC11658364; doi:10.1186/s13041-024-01166-7)

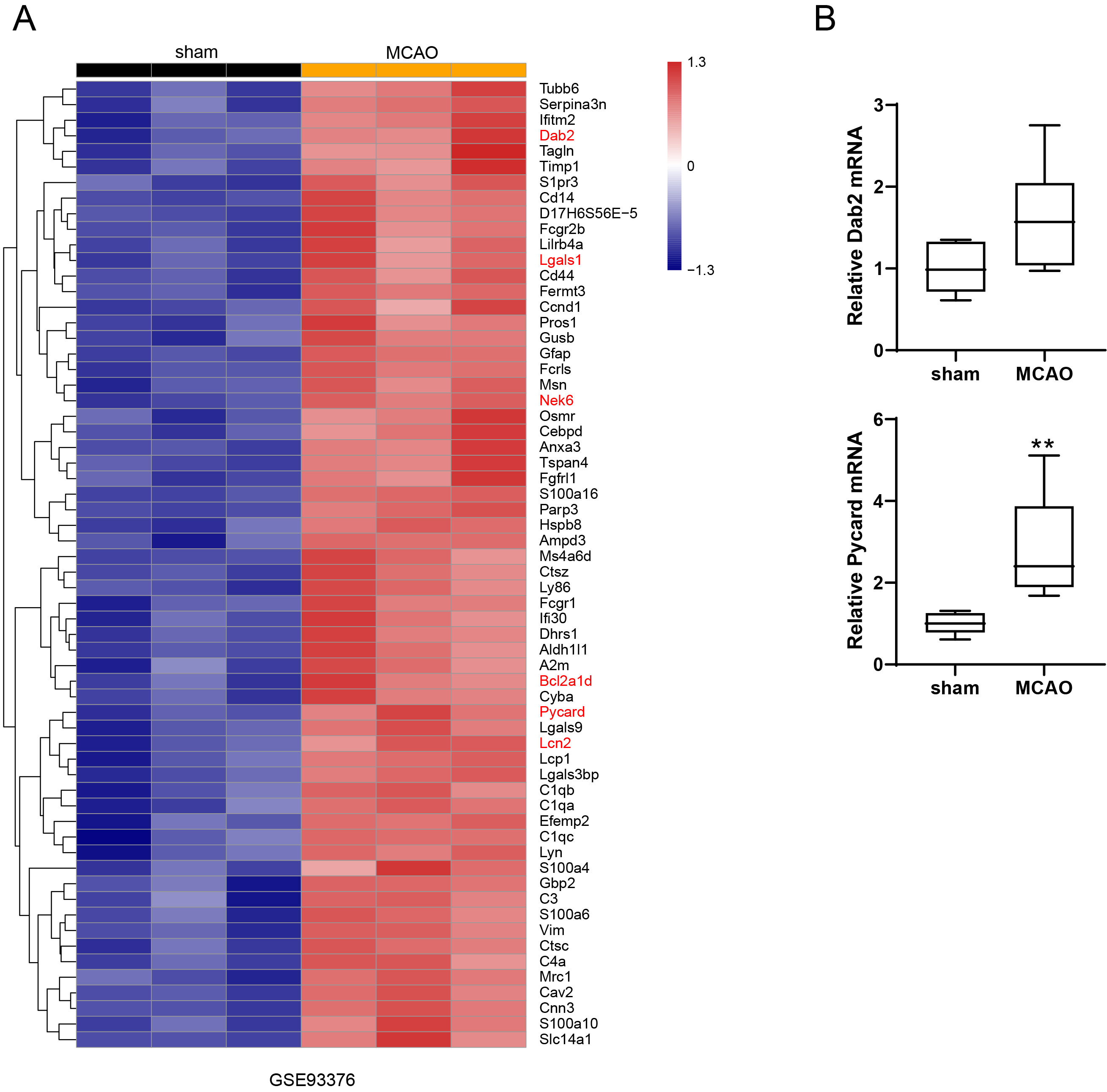

Supplement: Supplementary file 1 — Supplementary Material 1. Fig. 1 (A) Bioinformatics analysis was performed on dataset GSE93376 in GEO database. 62 differentially expressed genes were subjected to Gene Ontology functional annotation. B QPCR was used to detect the levels of Dab2 and Pycard. N = 6. Difference was calculated using unpaired two tailed T test. **P < 0.01 vs. sham group. [file 13041_2024_1166_MOESM1_ESM.jpg]

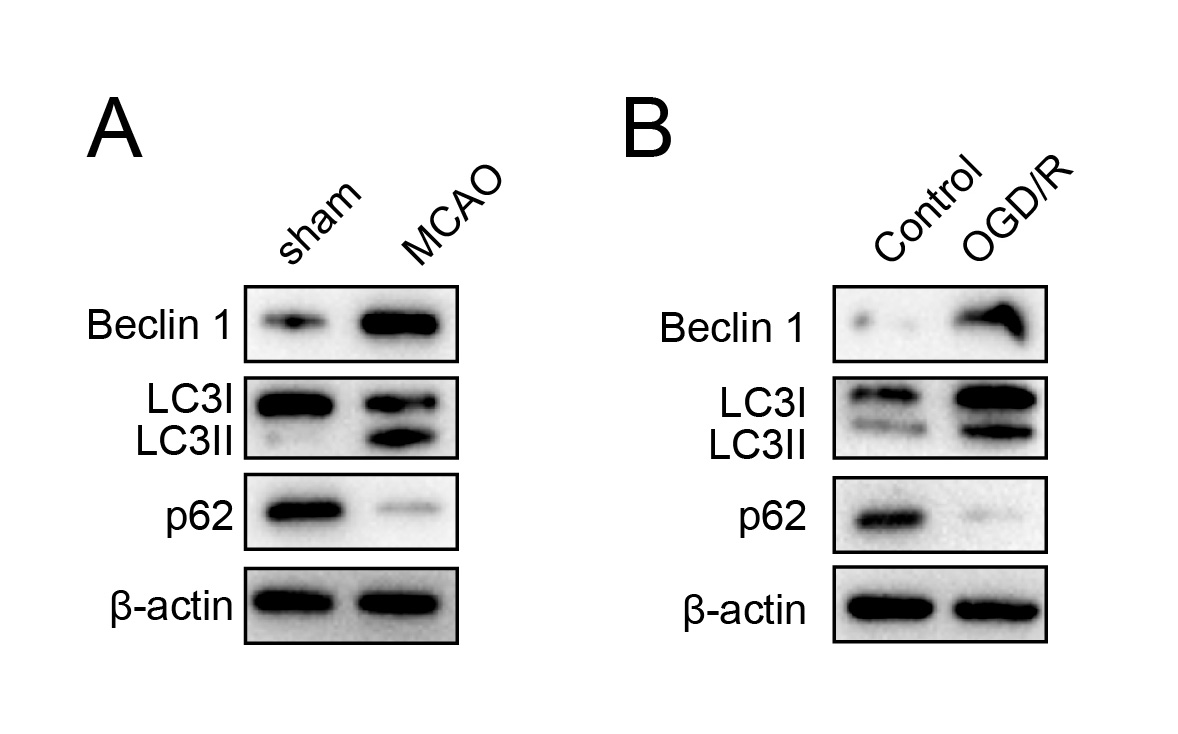

Supplement: Supplementary file 2 — Supplementary Material 2. Fig. 2 Western blot was used to detect the expressions of LC3 I/II, Beclin 1 and p62 in vivo (A) and in vitro (B). [file 13041_2024_1166_MOESM2_ESM.jpg]

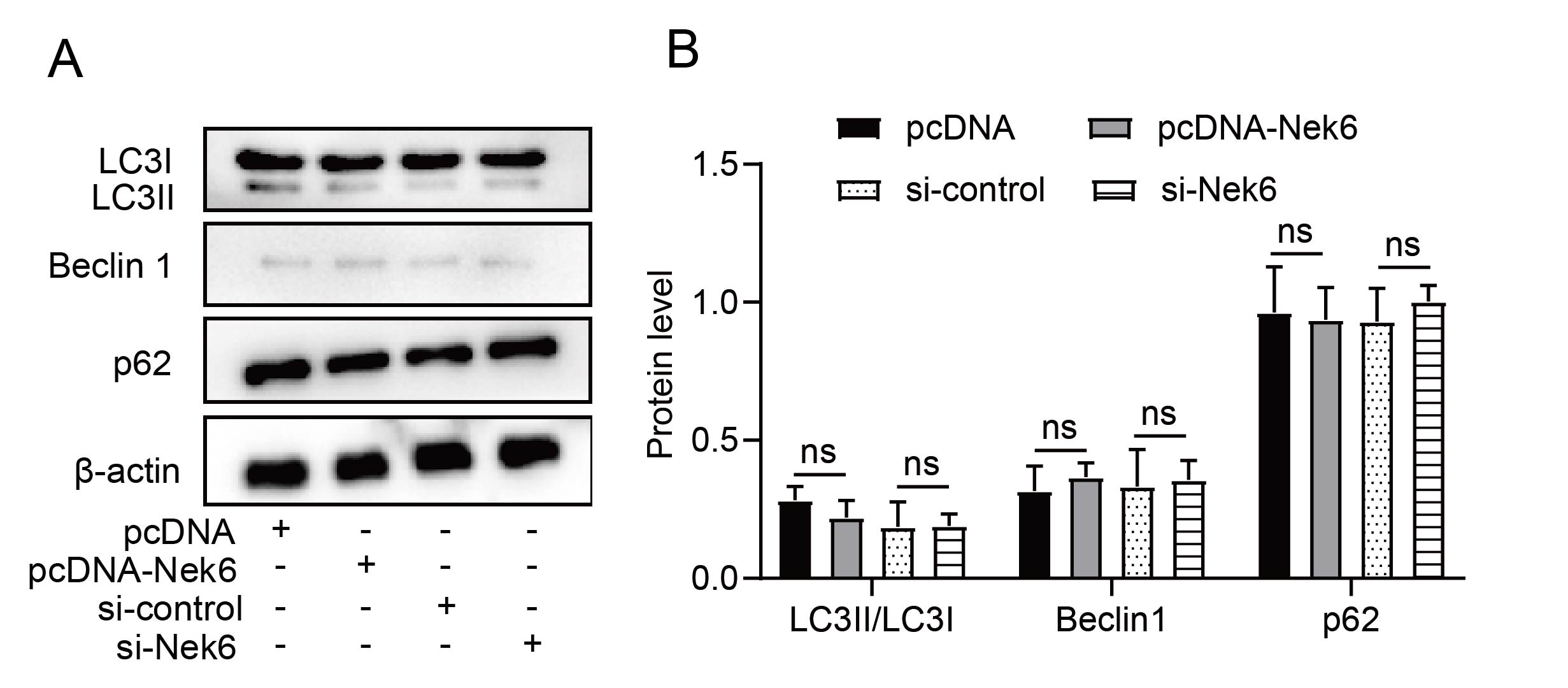

Supplement: Supplementary file 3 — Supplementary Material 3. Fig. 3 (A) SH-SY5Y cells were grouped into pcDNA, pcDNA-Nek6, si-control, si-Nek6. Western blot was used to detect the expressions of LC3 I/II, Beclin 1 and p62. Difference was calculated using unpaired two tailed T test. ns: no significance. [file 13041_2024_1166_MOESM3_ESM.jpg]

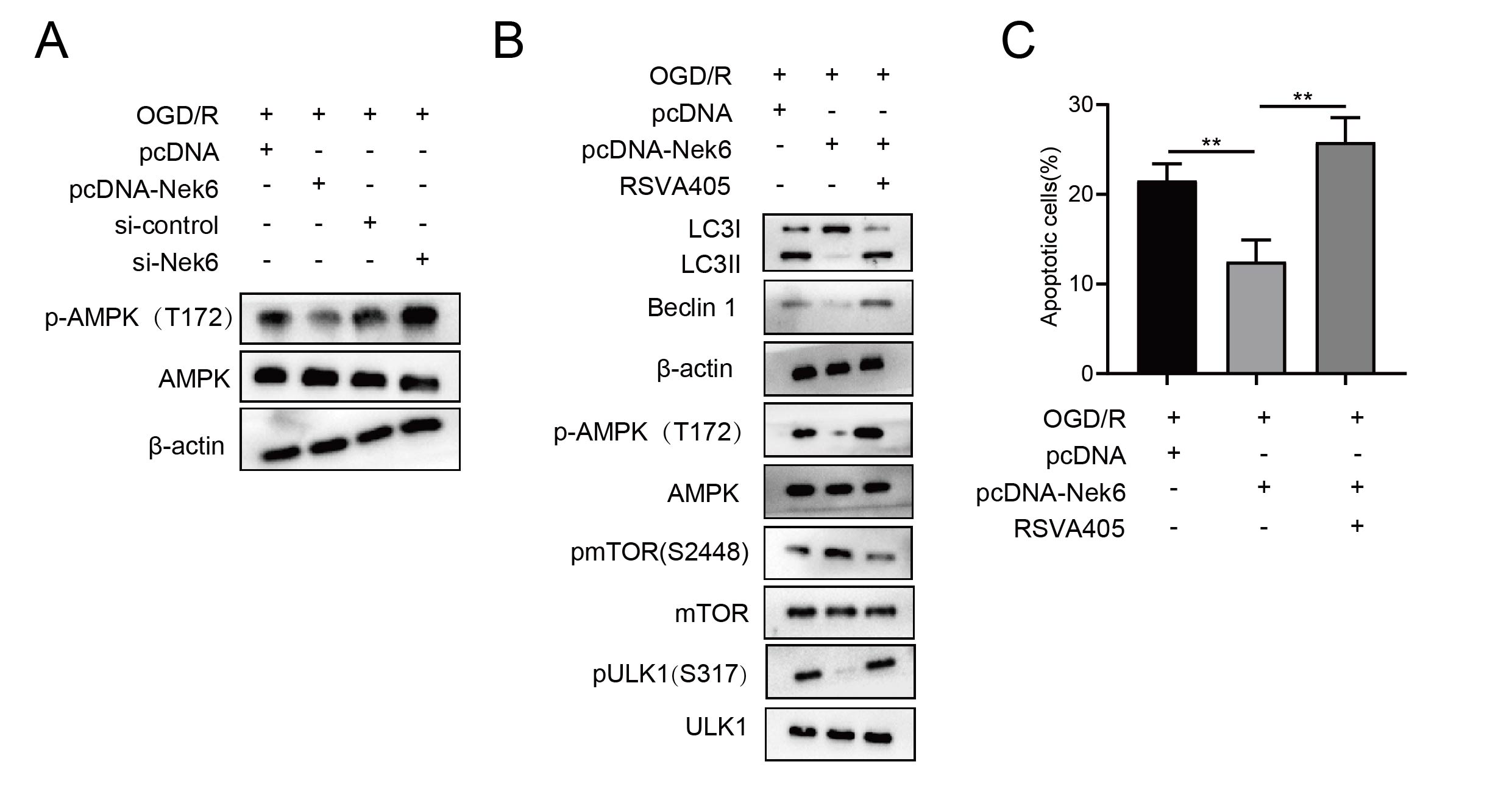

Supplement: Supplementary file 4 — Supplementary Material 4. Fig. 4 (A) SH-SY5Y cells were grouped into OGD/R + pcDNA, OGD/R + pcDNA-Nek6, OGD/R + si-control, OGD/R + si-Nek6. Western blot was used to detect the expressions of p-AMPK and AMPK. (B-C) SH-SY5Y cells were grouped into OGD/R + pcDNA, OGD/R + pcDNA-Nek6, and OGD/R + pcDNA-Nek6 + RSVA405. (B) Western blot was used to detect the expressions of LC3 I/II, Beclin 1, p-AMPK, AMPK, p-mTOR, mTOR, ULK1 and p-ULK1. (C) Cell apoptosis was detected by flow cytometry. Difference was calculated using one way anova with tukey’s post-hoc. **P < 0.01 vs. OGD/R + pcDNA or OGD/R + pcDNA-Nek6. [file 13041_2024_1166_MOESM4_ESM.jpg]

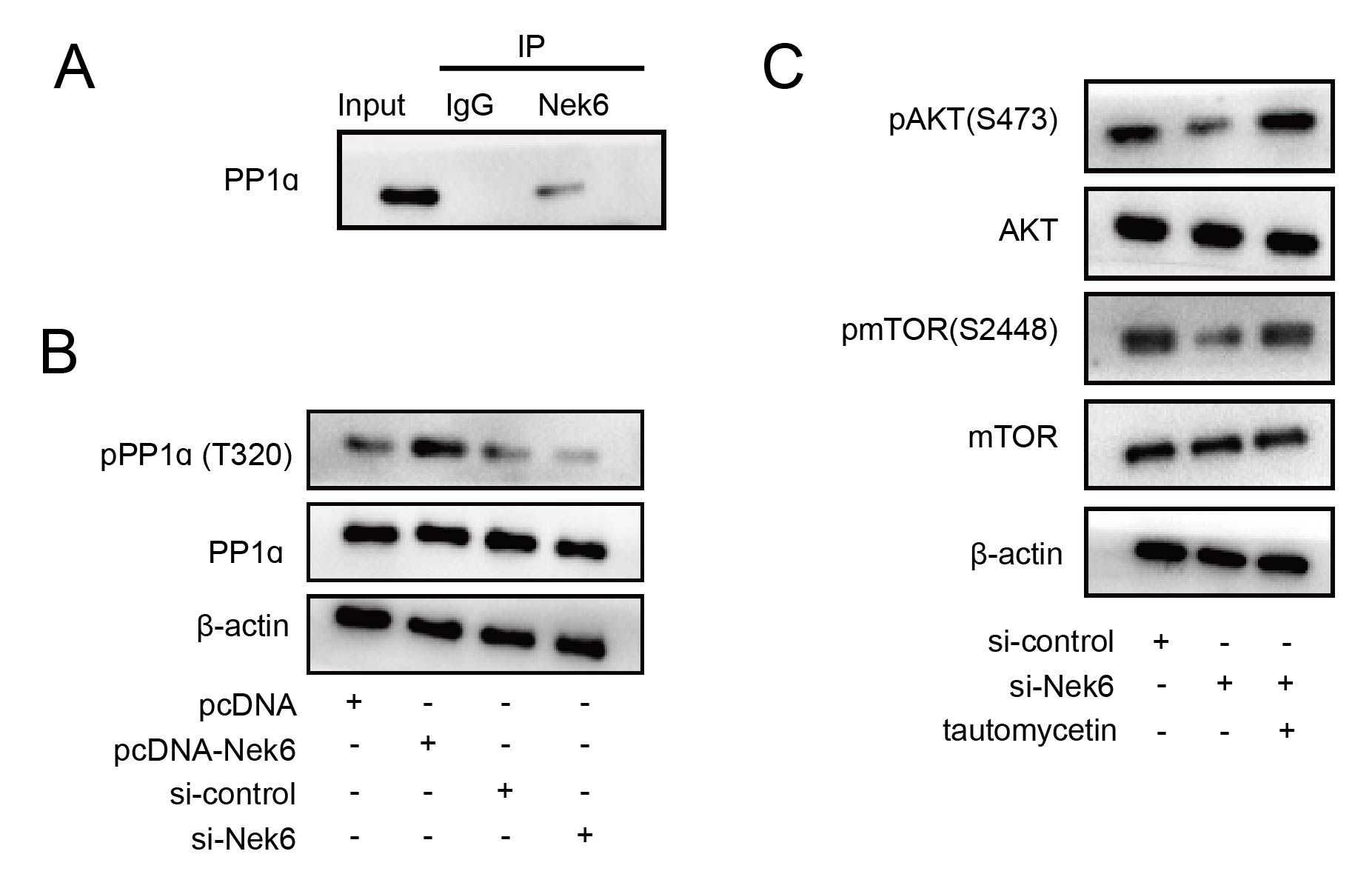

Supplement: Supplementary file 5 — Supplementary Material 5. Fig. 5 (A) The interaction between Nek6 and PP1 was validated by Co-IP assay. (B) Western blot was used to detect the expressions of PP1α, p-PP1α (T320) in the pcDNA, pcDNA-Nek6, si-control, si-Nek6 groups. (B) Western blot was used to detect the expressions of p-AMPK, AMPK, p-mTOR, mTOR in the si-control, si-Nek6, si-Nek6 + tautomycetin groups. [file 13041_2024_1166_MOESM5_ESM.jpg]
